# Severe Immune-Mediated Colitis Induced by Checkpoint Inhibitors in an Adolescent With Lynch Syndrome

**DOI:** 10.7759/cureus.43246

**Published:** 2023-08-09

**Authors:** Rachel V Lee, Kurt A Melstrom, Rifat Mannan, Gregory E Idos, Trilokesh Kidambi

**Affiliations:** 1 Center for Precision Medicine, City of Hope Comprehensive Cancer Center, Duarte, USA; 2 Department of Surgery, Division of Colorectal Surgery, City of Hope Comprehensive Cancer Center, Duarte, USA; 3 Department of Pathology, City of Hope Comprehensive Cancer Center, Duarte, USA; 4 Department of Internal Medicine, Division of Gastroenterology, City of Hope Comprehensive Cancer Center, Duarte, USA

**Keywords:** immunotherapy, pediatric oncology, colitis, immune checkpoint inhibitors, lynch syndrome

## Abstract

Lynch syndrome is a hereditary colorectal cancer caused by mutations in DNA mismatch repair genes. Immune checkpoint therapies have shown promise in treating Lynch syndrome-associated cancers but can lead to immune-related adverse events, such as colitis. In this report, we present a severe case of immune-mediated colitis (IMC) induced by checkpoint inhibitors in a young patient with Lynch syndrome. This 20-year-old male with Lynch syndrome and a history of glioblastoma underwent dual checkpoint therapy, after initial treatment with systemic steroids. Despite this, his condition worsened, resulting in complications, such as toxic megacolon and small bowel obstruction. He was subjected to various treatments, including infliximab and vedolizumab, but ultimately required total abdominal colectomy with J-pouch creation. This case highlights the challenges of managing severe IMC in patients with Lynch syndrome. The patient’s suboptimal response to standard treatments and the development of complications emphasizes the need for a better understanding and alternative therapeutic options for IMC. This case also calls into question whether a subset of patients with IMC should be “treated to target,” even though the current standard of care for IMC is guided by symptom response, and if so, further research is necessary to identify potential therapeutic targets. Further research is also required to understand the mechanisms of IMC and develop effective treatment strategies tailored to patients with Lynch syndrome and immune-related adverse events.

## Introduction

Although most colorectal cancers (CRCs) occur sporadically, inherited cancer syndromes or mutations cause approximately 5-10% of all known cases [1], with Lynch syndrome being the most common hereditary CRC syndrome (2-3% of the population) [2]. Lynch syndrome results from inherited mutations in DNA mismatch repair genes, leading to inadequate protein expression and function [1]. Over the years, there has been a marked increase (80%) in CRC cases and an increase in the lifetime risk of Lynch syndrome-associated cancers, such as endometrial cancer (60%). Therefore, identifying patients with Lynch syndrome, especially the proband in a family, can lead to early detection of individuals at risk and improve cancer monitoring and prevention [1]. Recently, immune checkpoint-based therapy has proven to be highly effective in solid tumors that are microsatellite unstable (MSI-high), irrespective of their origin [3]. Therefore, patients with Lynch syndrome are optimal candidates for immune checkpoint-based therapies [3].

Immune checkpoint inhibitors (ICI), including programmed cell death-1, programmed cell death-ligand 1, and cytotoxic T lymphocyte antigen 4 inhibitors, have various and unprecedented benefits in multiple malignancies. However, immune-related adverse events, such as colitis, are potential side effects [4]. Previous studies have shown that nearly 20-30% of patients develop diarrhea after ICI therapy, while no more than 5% of patients develop colitis [5]. Although the underlying mechanism of ICI-related colitis remains unknown, some possible mechanisms have been proposed, such as hyperactivation of effector T cells and infiltration of lymphocytes, causing a proinflammatory status and the emergence of autoimmune-type presentation [4].

Currently, the National Comprehensive Cancer Network guidelines state that for the management of severe colitis, immunotherapy should be discontinued, methylprednisolone should be administered intravenously in the inpatient setting, and biologic agents, such as infliximab (IFX) and vedolizumab, should be considered [6]. In this report, we describe a unique case of severe immune-mediated colitis (IMC) that was refractory to steroids and other biological immunosuppressive treatments and resulted in total abdominal colectomy with staged J-pouch creation in a patient with Lynch syndrome.

## Case presentation

**Figure 1:**
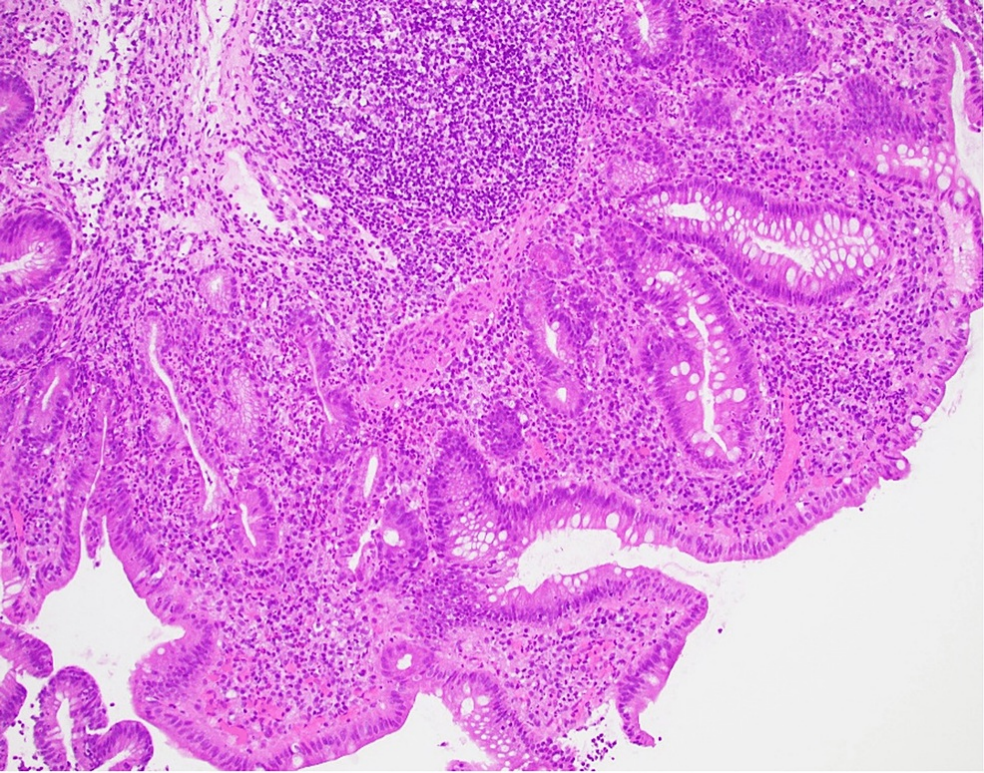
Colonic biopsy showing crypt architectural distortion, increased lamina propria inflammation and crypt abscess, based on hematoxylin and eosin (H&E) staining (magnification ×10).

**Figure 2:**
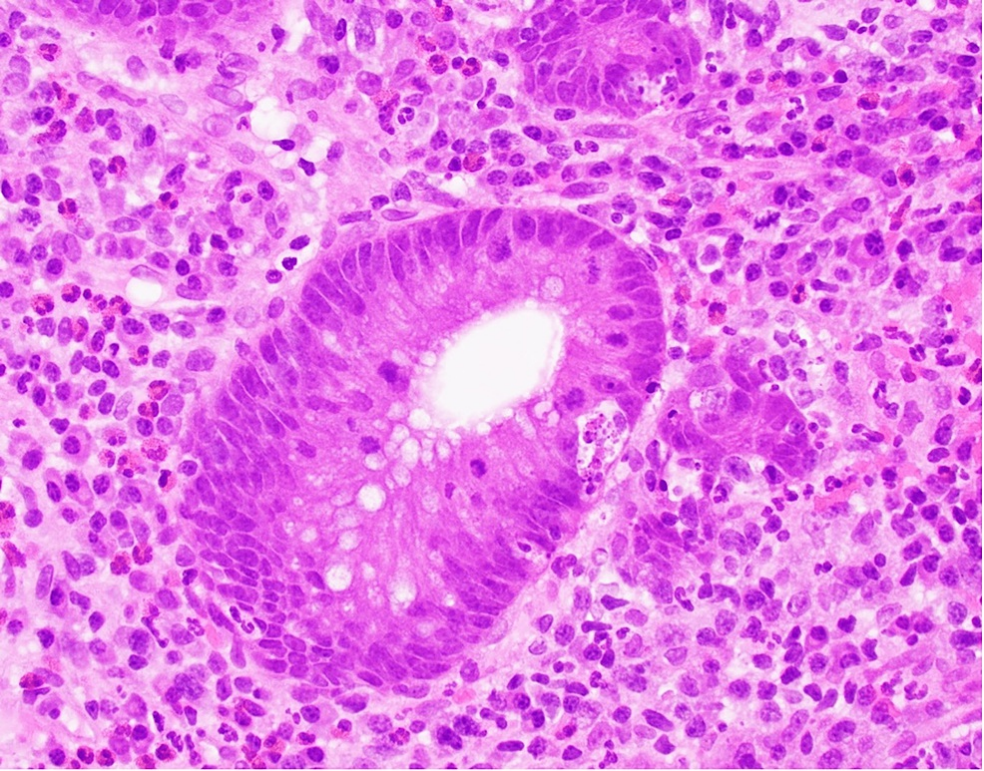
Higher magnification showing colonic crypt with crypt apoptosis, focal cryptitis, and reactive epithelial changes (hematoxylin and eosin (H&E) ×40).

A 20-year-old man with a medical history of Lynch syndrome who underwent glioblastoma multiforme resection in 2019 and subsequently received dual checkpoint therapy with pembrolizumab and nivolumab was presented with bloody diarrhea one year later, which was treated with oral systemic steroids for presumed IMC. However, progressive symptoms prompted an inpatient evaluation at a hospital, with laboratory results confirming anemia, elevated inflammatory markers, and fecal calprotectin. Initial colonoscopy revealed left-sided colitis. Microscopy revealed a chronic active colitis pattern of injury characterized by crypt architectural distortion, cryptitis, crypt abscesses, and an increase in crypt apoptosis. Overall, the histological findings were consistent with those of checkpoint inhibitor-associated colitis (Figures 1, 2). Additionally, the patient was negative for cytomegalovirus immunostaining.

**Figure 3:**
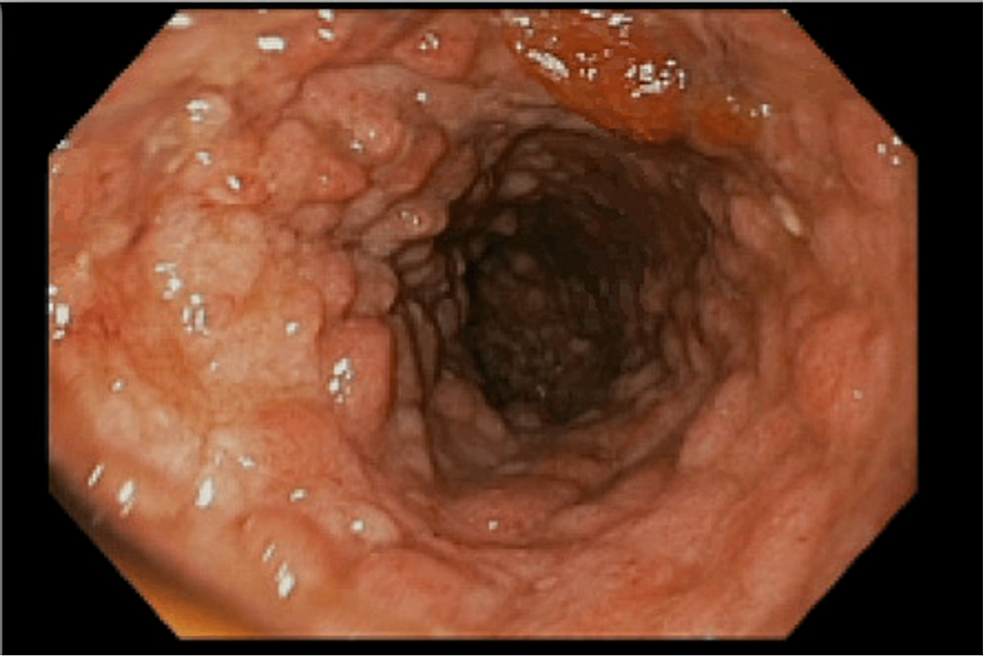
Sigmoidoscopy indicating severe pancolitis with ulceration and cobblestoning. There was also spontaneous bleeding with passage of the scope.

Considering the patient’s suboptimal response to steroids, infliximab (5 mg/kg) was administered, which improved abdominal pain and reduced bloody stools, and the patient was subsequently discharged with steroid tapering. However, the patient was readmitted one week later with diarrhea and decreased oral intake. Workup revealed hyponatremia and hypokalemia, and stool PCR positivity for both adenovirus and Clostridium difficile infections. Accordingly, the patient received vancomycin for the C. difficile infection and required total parenteral nutrition to manage severe malnutrition. The symptoms did not improve after the second dose of infliximab. Within days, the patient developed fever and abdominal distention, and computed tomography of the abdomen and pelvis showed significant distention of the transverse colon due to toxic megacolon. Thus, decompressive colonoscopy was performed, with improvement in symptoms and the return of bowel function. A follow-up test yielded negative results for C. difficile. Feeding was advanced, and total parenteral nutrition was discontinued. Routine laboratory examination prior to the third infliximab infusion (six weeks after the initial dose) showed an acute drop in hemoglobin levels, although the symptoms improved. The patient was administered an infusion and referred to our center for evaluation of refractory IMC. Restaging colonoscopy continued to show severe pancolitis, as shown in Figure 3, and laboratory tests showed an undetectable infliximab level with a high titer of anti-IFX antibodies. To diagnose IMC refractory to infliximab due to immunogenicity-induced secondary loss of response, the patient was started on vedolizumab (standard induction sequence of 300 mg intravenously at weeks 0, two, and six, and then assessed for doses every four weeks) along with a steroid tapering after improvement in symptoms.

**Figure 4:**
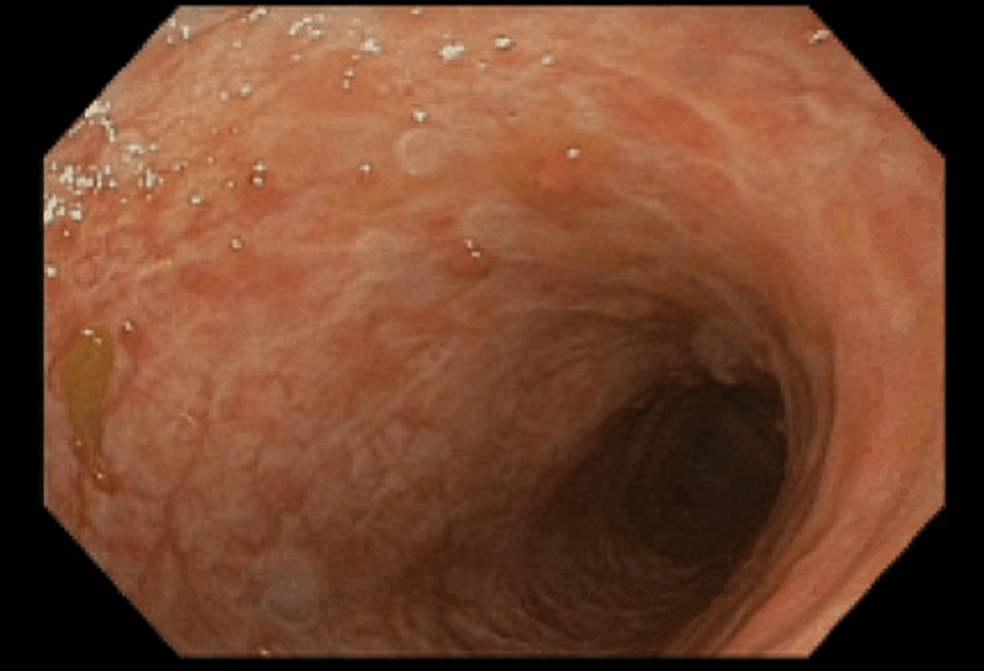
Immune-mediated colitis in the rectum improved after vedolizumab treatment. The friability, spontaneous bleeding, and cobblestone appearance resolved. There were no ulcerations, and only absent haustra and vasculature were observed.

After five doses of vedolizumab (three-dose induction sequence + two-dose intensified maintenance doses after a low vedolizumab trough of 5.4 µg/mL), ulceration size and depth showed a decrease on endoscopic evaluation, and there was an improvement in laboratory findings. However, a sixth dose of vedolizumab was administered due to mild symptoms. Restaging after a prolonged period of symptom resolution showed near-complete endoscopic remission (Figure 4). Accordingly, infusions were discontinued, and the patient continued steady recovery for three months before developing diarrhea and rectal bleeding. At the time of readmission, the patient was presented with severe abdominal pain, acute kidney injury, elevated levels of inflammatory markers, and colitis on computed tomography. Flexible sigmoidoscopy showed left-sided colitis. The vedolizumab level was rechecked and found to be 41 g/mL, with an anti-vedolizumab antibody level < 25. Steroids were re-induced, and an additional dose of vedolizumab was administered due to ongoing symptoms. A final attempt to avert surgery per the patient’s preference was undertaken in a trial of tofacitinib at 5 mg twice daily. Unfortunately, the symptoms were unresponsive to the medical therapy, given his history of Lynch syndrome. After multidisciplinary discussion, it was determined that a laparoscopic total abdominal colectomy with end ileostomy would be the best course of action, which the patient agreed to. The surgery was uncomplicated, and histological examination revealed a focal perforation with severely active erosive pancolitis.

Unfortunately, the patient developed a small bowel obstruction secondary to inflammation at the distal end of the ileostomy one month later. This admission culminated in an emergency overlap with small bowel resection, bowel decompression, lysis of adhesions, and ileostomy revision. The ileostomy excision pathology demonstrated severe erosive and focally ulcerating ileitis with architectural disorganization involving the transection margins. Once the disease stabilized, the patient underwent robot-assisted completion proctectomy and a J-pouch with diverting loop ileostomy was constructed. Pathological examination of the rectal specimen revealed severe erosive and focally ulcerating colitis with architectural disorganization and 15 pericolonic lymph nodes with reactive lymphoid hyperplasia. The patient had recently undergone ileostomy closure with no complications. Ileostomy pathology showed a segment of the small intestine with rare apoptotic figures involving intestinal crypts but was negative for dysplasia or malignancy. At six-month post-operative follow-up, pouchoscopy revealed no evidence of active inflammatory disease in the pouch or ileum.

## Discussion

Here, we present a unique case of severe IMC in a 20-year-old man with Lynch syndrome requiring IFX, with secondary loss of response due to immunogenicity and vedolizumab. The patient showed improvement, followed by a flare, despite high levels of the drug in his system. Although immunotherapy is a standard-of-care treatment option for individuals with Lynch syndrome and cancer, further research into other treatment options for IMC is necessary, given the potential non-responsiveness to standard-of-care treatments, such as vedolizumab. Additionally, an improved understanding of ICIs and common gastrointestinal side effects is necessary, as symptom recognition is vital for treatment and outcomes.

Patients with IMC usually present with diarrhea, cramping abdominal pain, and rectal bleeding. Generally, physical examination results range from normal to abdominal tenderness and distention. However, the presence of other examination signs, including guarding, rigidity, or rebound tenderness, suggests complications, such as colonic perforation or toxic megacolon [4].

The incidence of IMC also varies based on drug and dose exposure and can occur anywhere between two and 24 months after treatment initiation, and early endoscopic evaluation is recommended for diagnostic and prognostic assessments. Infliximab is indicated for steroid-refractory cases. Most patients require a single dose, although a second or third dose is also commonly required. Recent studies have found that higher initial steroid doses are associated with lower survival rates. Therefore, if these results are continuously validated, they would support a lower threshold for starting infliximab, with less reliance on steroids [7]. The use of vedolizumab, a monoclonal antibody that targets integrin a4B7, has also been described in adults. The present case is unique because the patient initially responded both clinically and endoscopically to vedolizumab and later developed a flare without rechallenge with immunotherapy. Although “treatment to target” is the standard of care for inflammatory bowel disease (IBD) management, it is not the standard management strategy for IMC. In IMC, treatment is guided by the symptom response; therefore, endoscopic or histological staging of the disease is uncommon. However, the patient’s symptoms improved in this case, steroids were discontinued, and his endoscopic disease also ameliorated. Overall, this case calls into question whether a subset of patients with IMC should be “treated to target,” and if so, further research is necessary to identify potential therapeutic targets. IMC can be recurrent and aggressive, and current medical treatments may not completely induce remission. The surgical history of our patient, which resulted in total abdominal colectomy with end ileostomy complicated by bowel obstruction with ileostomy revision, J pouch and proctectomy, and ileostomy closure, was extensive and anxiety-inducing. Therefore, it is critical to acknowledge the potential severity and consequences of IMCs.

Whether checkpoint therapy can unmask/induce IBD was also considered; however, the pathological findings did not support this hypothesis. Immunotherapy-related colitis is characterized by a spectrum of morphological findings on colonic biopsy, including apoptotic colopathy, lymphocytic colitis/collagenous pattern, focal/diffuse active colitis, and chronic active colitis (often a combination of patterns is observed). Endoscopic presentation varies from mild to moderately severe inflammation with ulcers and exudates, and to severe inflammation with deep ulcerated mucosa [8]. Colonic biopsies showed varying degrees of chronic active colitis with increased crypt apoptosis; however, such changes can also be encountered in patients with IBD. Given the patient’s clinical history, including immunotherapy, the results are most likely secondary to immunotherapy-induced colonic mucosal injury, and the symptoms are unlikely to be attributed to IBD.

For patients who achieve symptomatic remission, the decision to reintroduce ICI therapy is individualized and requires a multidisciplinary approach with input from oncology and gastroenterology specialists [4]. Further research is required to better understand the pathophysiology and risk factors of IMC.

## Conclusions

This case is a compelling report of immune-mediated colitis in an adolescent with Lynch syndrome who underwent dual checkpoint therapy with pembrolizumab and nivolumab for glioblastoma. Unfortunately, the patient did not respond well to standard-of-care treatments and developed severe complications, underscoring the challenges in managing IMC in the context of Lynch syndrome. This case also raises important questions regarding the potential for a “treat to target” approach for IMC and the need for alternative therapeutic options in refractory cases. It also indicates the need for further advancements in research to better understand the mechanisms by which IMC occurs in order to develop strategies and treatments to target immune-related adverse events. Ultimately, the findings from this case emphasize the significance of early recognition and timely intervention to improve outcomes in patients with Lynch syndrome who receive immune checkpoint therapies.
